# Enhancement in Photovoltaic Performance of Solar Cells by Electrostatic Adsorption of Dyes on ZnO Nanorods

**DOI:** 10.3390/nano12030372

**Published:** 2022-01-24

**Authors:** Seong Il Cho, Baekseo Choi, Byeong Chul Lee, Yunsung Cho, Yoon Soo Han

**Affiliations:** 1 School of Advanced Materials and Chemical Engineering, Daegu Catholic University, Gyeongbuk 38430, Korea; pokw1231@naver.com (S.I.C.); skdiwlsdnr@naver.com (B.C.); bclee@live.co.kr (B.C.L.); 2 School of Electronic and Electrical Engineering, Daegu Catholic University, Gyeongbuk 38430, Korea; philos@cu.ac.kr

**Keywords:** dye-sensitized solar cell, ZnO nanorod, chemical bath deposition, electrostatic dye adsorption

## Abstract

ZnO nanorods were formed by chemical bath deposition on fluorine–doped tin oxide (FTO) glass and the photovoltaic performance of ZnO-based dye-sensitized solar cells (DSCs) was investigated. A DSC with 8 h-grown ZnO nanorods showed a higher power conversion efficiency (PCE) than devices with 4, 6, and 10 h-grown ones. Further improvement in PCE was achieved in a cell with a silver-ion-deposited ZnO/FTO electrode. By deposition of Ag^+^ on the surface of the 8 h-grown ZnO nanorods, the dye-loading amount increased by approximately 210%, compared to that of pristine ZnO nanorods, resulting in a 1.8-times higher PCE. A DSC with the pristine ZnO/FTO electrode showed a PCE of 0.629%, while in a device with the silver-ion-deposited ZnO/FTO, the PCE increased to 1.138%. In addition, interfacial resistance at the ZnO/dye/electrolyte was reduced to approximately 170 Ω from 460 Ω for the control cell with the pristine ZnO/FTO. We attributed the higher dye-loading amount in the silver-ion-deposited ZnO/FTO to the electrostatic attraction between the positively charged ZnO and carboxylate anions (–COO^−^) of the N719 dyes.

## 1. Introduction

Dye-sensitized solar cells (DSCs) are among third-generation photovoltaic cells and have several attractive features. They can be semi-flexible and semi-transparent, and usually low cost; these properties enable their use in situations glass-based solar cells cannot be used [1,2]. In addition, they have low fabrication cost, fast assembling process, and low toxicity, and power conversion efficiencies (PCEs) of DSCs are constantly improved [3]. The PCE of a cell is given as the ratio of the maximum power (*P_max_*) to the total intensity of the incident light (*P_in_* = 100 mW/cm^2^). The fill factor (*FF*) of the cell is defined as the ratio of its *P_max_* to the product of short circuit current density (*J_sc_*) and open-circuit voltage (*V_oc_*), which leads to PCE = (*J_sc_* ·*V_oc_*·*FF*)/*P_in_* [where *FF* = *P_max_*/(*J_sc_*·*V_oc_*)] [4]. A general DSC consists of a glass substrate coated with fluorine-doped tin oxide (FTO), mesoporous TiO_2_ layer, light absorbing dye, Pt counter electrode, and an I^−^/I_3_^−^ electrolyte [5]. The mesoporous TiO_2_ layer as an n-type semiconductor is the acceptor of electrons injected from photoexcited dyes and provides a conductive pathway from the electron injection site to the FTO layer. Although the highest PCE of 14.3% has been achieved in a TiO_2_-based DSC [6], an additional enhancement in conversion efficiency is needed to compete with silicon or calcogenide semiconductor-based solar cells. However, TiO_2_-based DSCs are suffered from energy loss due to back electron transfer. This back electron transfer between I_3_^−^ in the electrolyte (or oxidized dye molecules) and electrons in the TiO_2_ conduction band is attributed to the lack of a depletion layer on the TiO_2_ surface [7].

As another n-type semiconductor, zinc oxide (ZnO), has an energy gap of 3.3 eV, which is similar to that of TiO_2_ (3.2 eV) and has a much higher electron diffusivity than TiO_2_. It also has a high electron mobility of 115–155 cm^2^ V^−1^ s^−1^, which facilitates electron transport in the semiconductor and the reduction of the back electron transfer. In addition, ZnO can be embodied with diverse morphologies such as nanorods, nanoflakes, nanorings, nanofibers, nanowires, nanosheets, or nanoflowers, which are due to ease of crystallization and anisotropic growth unlike the crystalline structure of TiO_2_ [7,8,9]. This fact can lead to various designs of photoelectrodes. Thus, since the first report on ZnO-based DSC [10], extensive research has been conducted to realize high performance of DSCs with it as a photoelectrode. Although much lower PCEs of 0.4–7.5% are recorded in the ZnO-based cells, compared to those of the TiO_2_ based ones, ZnO is still considered the best substitute for TiO_2_ in DSCs [7,8,9]. Major limitation of ZnO-based solar cells is the instability of ZnO in acidic dye, i.e., the Zn^2+^/dye complex is formed during the prolonged sensitization process. As shown in Figure S1, N719 dye [C_58_H_86_N_8_O_8_RuS_2_; di-tetrabutylammonium cis-bis(isothiocyanato)bis(2,2′-bipyridyl-4,4′-dicarboxylato)ruthenium(II)] with two strong visible absorption bands centered at 384 and 525 nm has carboxylic acid groups as an anchor on the surface of TiO_2_ or ZnO, and releases protons when it dissolves in solvents. In the fabrication process of ZnO-based DSCs, ZnO layer as an n-type semiconductor is dipped into a solution of acidic dye as a p-type semiconductor to form a pn junction. However, when ZnO is contact with acidic dye, protons from the dyes cause the dissolution of Zn atoms at ZnO surface, resulting in the formation of excessive Zn^2+^/dye complex, which lowers the electron injection efficiency from the excited dye to ZnO [7,8,9]. Thus, to increase the photovoltaic properties of ZnO-based solar cells, it is necessary to reduce the formation of the Zn^2+^/dye complex. In other words, dyes should be loaded on ZnO surfaces as much as possible in a short sensitization (dipping) time, usually 20 min, to reduce the complex formation.

Ko et al. demonstrated that the H^+^-deposited TiO_2_ electrode induced high electrostatic attraction between the positively charged TiO_2_ and the anions of the dyes, leading to a much faster sensitization rate [11]. Meanwhile, it has been reported that metal ions such as Pb^2+^, Cd^2+^, Cu^2+^, and Ag^+^ can be removed from their solutions by contact with ZnO nanoparticles via a mechanism of adsorption, reduction, and/or oxidation [12,13,14]. This indicates that metal ions can be adsorbed on the surface of the ZnO particles. DSCs with Ag-decorated ZnO, Ag/ZnO composite or Ag-doped ZnO have been reported [15,16,17,18,19,20], and the Ag incorporation on ZnO layers resulted in an enhancement in PCEs of ZnO-based solar cells.

In this study, an electrostatic method was applied to increase the dye-loading amount in a short sensitization time (20 min). ZnO nanorods were first formed by chemical bath deposition on FTO glass to produce ZnO/FTO photoelectrodes. Silver ions were deposited on the surface of the ZnO nanorods and then sensitized with N719 dyes. The resulting electrodes were used as the working electrodes of DSCs, and their photovoltaic performance was compared to that of control cells with conventionally sensitized ZnO layers. To the best of our knowledge, electrostatic dye adsorption on ZnO nanorods, which can lead to a considerable enhancement in the PCE of cells, has never been reported. Several methods for increasing dye-loading amount and their effects are summarized in Table 1, including those of our device with the silver-ion-deposited ZnO layer.

## 2. Materials and Methods

### 2.1. Materials

To grow ZnO nanorods and fabricate DSCs, the same materials as those used in our previous work were exploited [25]. Detailed materials information is provided in the electronic supplementary information (ESI). Silver (I) nitrate (AgNO_3_) from Daejung Chemicals and Metals Co., Ltd. (Gyeonggi-do, Korea) was used as a surface modifier. ZnO powder was purchased from Kojundo Chemical Laboratory Co., Ltd. (Saitama, Japan).

### 2.2. Preparation of ZnO-Based DSCs

Except for the deposition of silver ions onto the ZnO nanorod surface, the same procedures described in our previous work [25] were applied to prepare ZnO-based DSCs. The deposition process of silver ions is as follows. The FTO glasses coated with ZnO nanorods (i.e., ZnO/FTO photoelectrodes) were soaked in an aqueous solution (50 mM) of silver nitrate for 0–40 min to deposit silver ions onto the ZnO surfaces. Subsequently, the resulting electrodes were rinsed with water and ethanol, and then dried at 65 °C for 10 min to produce silver-ion-deposited electrodes (Ag^+^–ZnO/FTO). The pristine ZnO/FTO and Ag^+^–ZnO/FTO photoelectrodes were separately immersed into 0.5 mM of ethanolic N719 dye solution for 20 min to form a pn junction, followed by rinsing and drying to produce the working electrodes. Detailed fabrication conditions of ZnO-based DSCs are provided in the ESI. An energy band diagram of the fabricated ZnO-based DSC is illustrated in Figure S2 of the ESI [26,27].

### 2.3. Measurements

We performed morphology observations and elemental analyses of ZnO nanorods formed on FTO glass. Absorptiometric analysis was conducted to quantify amounts of N719 dyes adsorbed on ZnO nanorods. Photovoltaic performance, open-circuit voltage decay (OCVD) measurements and electrochemical impedance spectroscopic (EIS) analyses of ZnO-based DSCs were carried out. Detailed information for the measuring instruments is presented in the ESI.

## 3. Results

### 3.1. Optimal Growth Time of ZnO Nanorods

**Figure 1:**
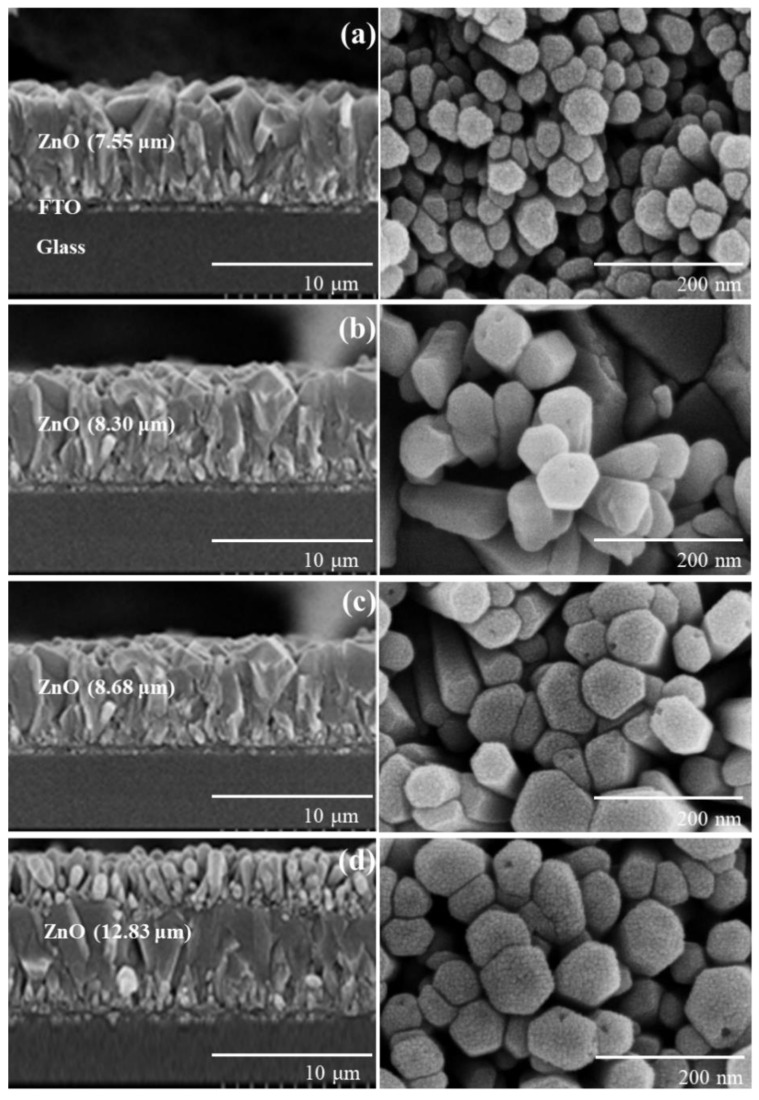
Cross-sectional (**left**) and top view (**right**) SEM images of ZnO nanoroads formed on FTO glasses for (**a**) 4, (**b**) 6, (**c**) 8, and (**d**) 10 h growth time.

The ZnO nanorods formed on FTO glass by chemical bath deposition (CBD) are visualized in Figure 1. To provide more clear morphology of the 8 h-grown ZnO nanorod, a SEM image with different magnification was presented in Figure S3 of the ESI. We could fabricate vertically aligned ZnO nanorods by adjusting the growth temperature to 75 °C. With increasing growth time (4, 6, 8, and 10 h), the ZnO thickness increased from 7.6 to 12.8 μm. When the growth time was extended to 10 h, two-layered ZnO nanorods were built, as shown in Figure 1d. Similar phenomena were reported [28,29,30]. Long growth time of over 10 h destabilizes crystallographic plane (001) of ZnO nanorods, which is probably due to reduced concentration of Zn precursor, and thus new nuclei are formed on the (001) plane. This leads to double-layered ZnO structures. The XRD pattern of the ZnO/FTO electrode is shown in Figure S4 of the ESI. Characteristic peaks with the hexagonal wurtzite structure of ZnO were detected, and they were in good agreement with the JCPDS file 36–1451 [26,31]. As a reference, in our previous work, mixed ZnO layers of nanoflowers and microrods were formed at an elevated temperature of 90 °C and showed a randomly distributed morphology rather than a vertically aligned one [25].

**Figure 2:**
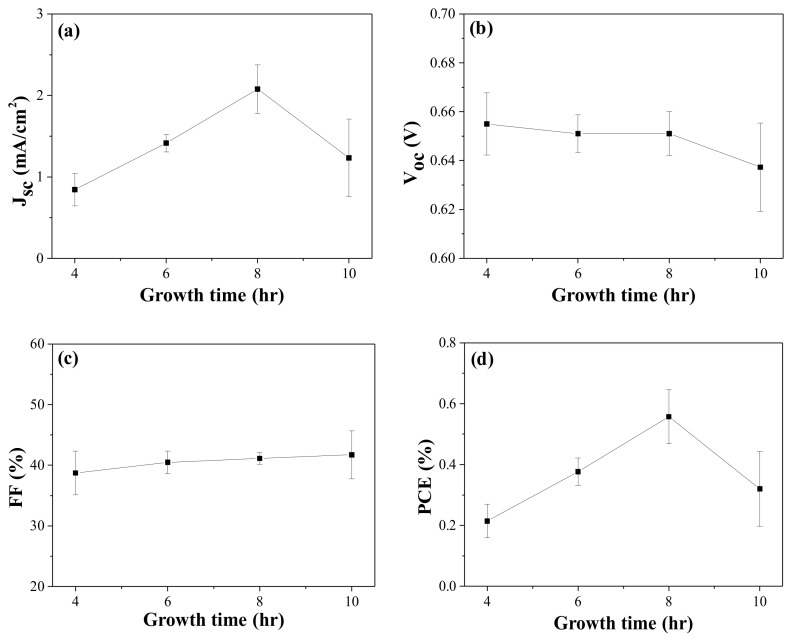
Photovoltaic performance variations with growth time in the CBD solution: (**a**) *J_sc_*, (**b**) *V_oc_*, (**c**) *FF*, and (**d**) PCE of the DSCs. *J_sc_*, *V_oc_*, and *FF* are measured under AM 1.5 condition.

Four DSCs in each condition were prepared using ZnO/FTO electrodes, and the influence of the growth time on the photovoltaic properties was examined. Figure 2 shows the averaged cell performance with the growth time, and the photovoltaic parameters are summarized in Table 2 and Table S1 of the ESI. An increment in the *J_sc_* was observed with increasing growth time from 4 to 8 h, whereas the *V_oc_* value was slightly decreased. There were no meaningful variations in the *FF* value with the growth time. As a result, a higher average PCE value was recorded at a growth time of 8 h. To confirm the influence of the growth time on the photovoltaic performance, we quantified the amount of dye loaded on the ZnO nanoroad surface on the basis of the Beer-Lambert equation and the absorbance of dyes detached from the ZnO surface [4,32]. The average dye-loading amounts, as estimated using four pristine ZnO/FTO photoelectrodes, increased with increasing growth time from 4 to 8 h (Table 2 and Table S2 of the ESI). Dependence of growth time on the density of the ZnO nanostructures was reported, i.e., by extending the growth time, the length of the nanorods increased, but the density decreased due to the coalescence and their faster growth toward the c-axis direction [33,34,35]. This can lead to a larger surface area of ZnO nanorods in 4–8 h of the growth time, and therefore to an increase in dye-loading amount. However, when the growth time reached 10 h, the loaded dyes were reduced, probably because of the two-layered ZnO nanorods. It was believed that dye molecules could not permeate the lower layer of the 10 h-grown ZnO nanorods. Overall, we attributed the higher PCEs in the DSCs with 8 h-grown ZnO/FTO to higher dye-loading amounts.

### 3.2. Deposition of Silver Ions on the ZnO Nanorod Surface

**Figure 3:**
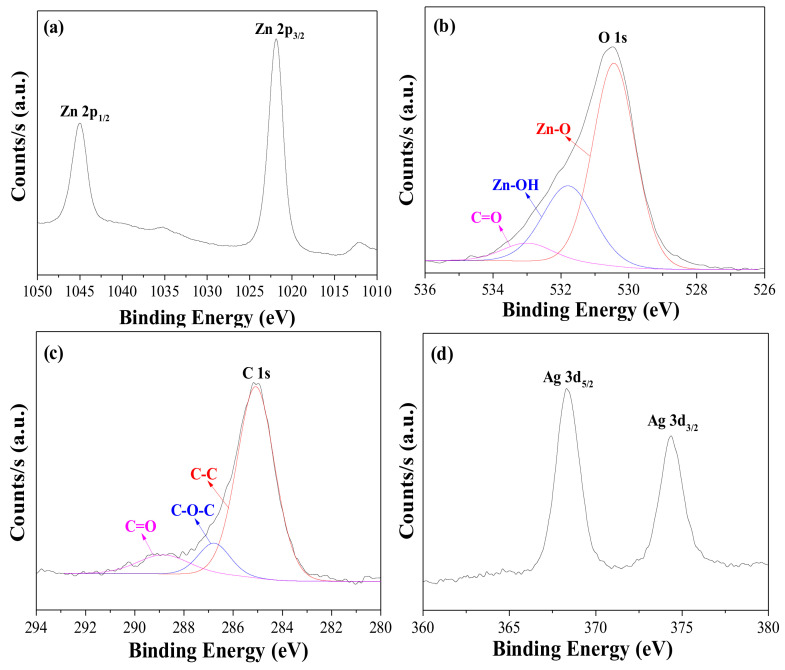
XPS spectra for (**a**) Zn 2p, (**b**) O 1s, (**c**) C 1s and (**d**) Ag 3d of Ag^+^(20)–ZnO/FTO electrode.

As mentioned earlier, metal ions can be adsorbed on the surface of the ZnO particles [12,13,14]. To deposit the silver ions on the ZnO nanorod surface, the 8 h-grown ZnO nanorods on FTO glass were immersed in an aqueous AgNO_3_ solution, rinsed with water, and dried. The deposition period was altered from 10 to 40 min to yield Ag^+^(10, 20, 30, 40)–ZnO/FTO, where “(20)” implies that the deposition period was 20 min. XPS measurements verified the implantation of silver ions on the ZnO surface. Figure 3 shows the XPS spectra for Zn, O, C and Ag of the Ag^+^(20)–ZnO/FTO, and its full-survey-scan spectrum is presented in Figure S5 of the ESI. Peaks were observed at 1045.0 and 1021.9 eV, which correspond to the binding energies of Zn 2p_1/2_ and 2p_3/2_, respectively (Figure 3a), and around 23.1 eV of the binding energy difference was consistent with the reference value [31,36]. As shown in Figure 3b, the O 1s region can be deconvoluted into three peaks, i.e., 530.4, 531.8 and 533.0 eV attributed to the Zn–O, Zn–OH (in oxygen deficient parts) and C=O (associated with C contamination), respectively. As an impurity, the C 1s peak was also found, and its spectrum can be fit into three peaks, corresponding to C–C, C–O–C and C=O bands at 285.1 eV, 286.8 eV and 288.9 eV, respectively. These detected peaks for Zn, O and C were well consistent with reported values [31,36]. As displayed in Figure 3d, the XPS peaks detected at 368.3 and 374.3 eV agreed with the binding energies of Ag 3d_5/2_ and Ag 3d_3/2_, respectively [37]. The N 1s peak (around 400 eV) originating from nitrate ions (NO_3_^–^) was not detected. It was thus evident that silver ions were deposited by simple soaking in an aqueous AgNO_3_ solution on the surface of 8 h–grown ZnO nanorods.

### 3.3. Photovoltaic Performance of DSCs with Ag^+^–ZnO/FTO

#### 3.3.1. Performance Variations with Deposition Time of Silver Ions

**Figure 4:**
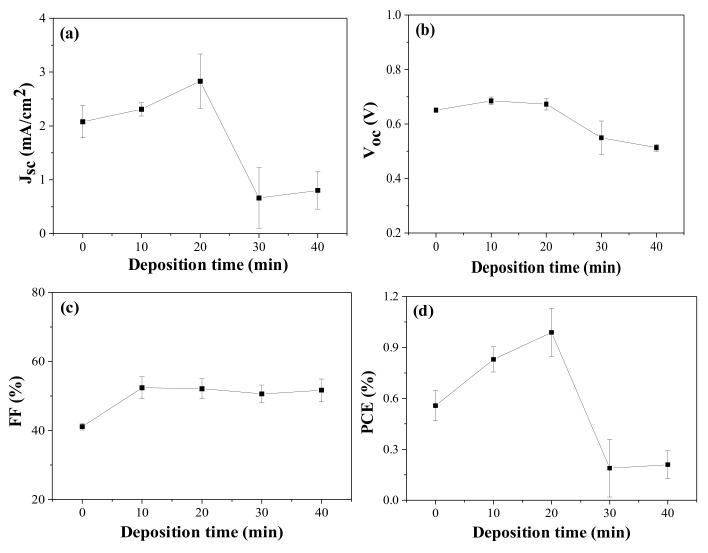
Photovoltaic performance variations with deposition period of silver ions: (**a**) *J_sc_*, (**b**) *V_oc_*, (**c**) *FF*, and (**d**) PCE of the DSCs with 8 h-grown ZnO nanorods. *J_sc_*, *V_oc_*, and *FF* are measured under AM 1.5 illumination.

It was reported that an improved PCE was achieved when Ag-incorporated ZnO was applied to DSCs [15,16,17,18,19,20]. In those reports, Ag was used in the form of nanoparticle (metal) or as a dopant. However, in this study, silver was used in the form of the ion to produce positive charge on the ZnO nanorod surface, and therefore to induce the electrostatic attraction between the positively charged ZnO and carboxylate anions (–COO^−^) of the N719 dyes. We prepared silver-ion-deposited electrodes [Ag^+^(10, 20, 30, 40)–ZnO/FTO] using 8 h-grown ZnO/FTO, and then DSCs with them were fabricated. The photovoltaic performance was compared to that of a control device with pristine ZnO nanorods grown for 8 h on FTO glass. The resulting performance of the DSCs with the deposition period of silver ions are presented in Figure 4, and Table 3 and Table S3 of the ESI. As can be seen in Figure 4a,b, the *J_sc_* and *V_oc_* values were improved as the deposition time was lengthened by 20 min, compared to those of the control device without silver ions. However, a sharp decrease in both *J_sc_* and *V_oc_* was observed after that time. An increment in the *FF* occurred by the deposition of silver ions on the ZnO surface. The overall efficiencies of the DSCs with Ag^+^(20)–ZnO/FTO were improved relative to those of the control cells with pristine ZnO/FTO electrodes.

**Figure 5:**
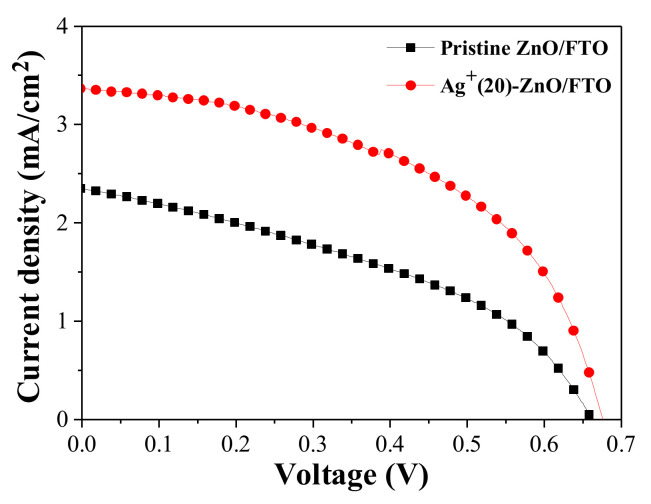
*J–V* characteristics of the DSCs with the pristine ZnO/FTO and Ag^+^(20)–ZnO/FTO photoelectrodes, measured under AM 1.5 condition.

When the deposition time of silver ions was 20 min, the highest PCE value was recorded, and thus we focused on this device with the Ag^+^(20)–ZnO/FTO to reveal the root of the improved efficiency. Figure 5 represents the current density (*J*) and voltage (*V*) curves of the champion DSCs with the pristine ZnO/FTO and Ag^+^(20)–ZnO/FTO, and the cell performance is listed in Table 4. The champion cell with the Ag^+^(20)–ZnO/FTO exhibited a PCE value of 1.138%, which was higher than that of the control cell with the pristine ZnO/FTO electrode (0.629%). The improved PCE was ascribed to an increase in the *J_sc_*, *V_oc_*, and *FF*. It is, therefore, worth investigating how such a simple deposition of silver ions on the ZnO surface increases the DSCs’ performance.

#### 3.3.2. Influences of Ag^+^ Deposition on the *J_sc_*

**Figure 6:**
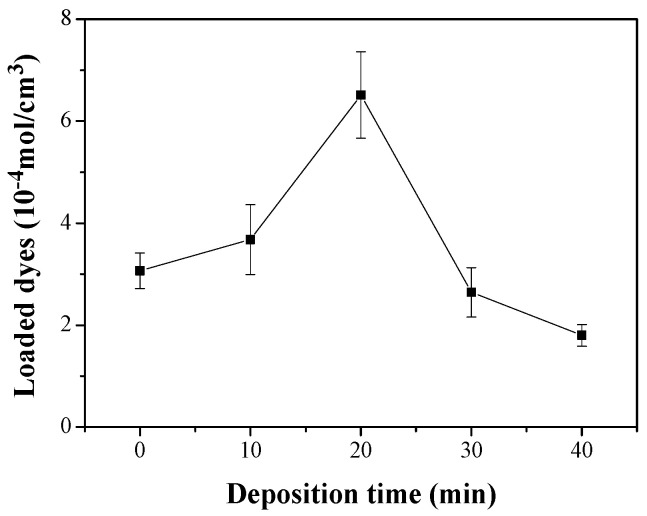
Amounts of N719 dyes as a function of deposition time of silver ions; where N719 dyes adsorbed on the ZnO nonorod surface were detached using an aqueous NaOH solution, and used in absorbance measurements.

The *J_sc_* value of the DSC with the Ag^+^(20)–ZnO/FTO increased by 3.362 mA/cm^2^ compared with that of the control cell with the pristine ZnO/FTO (2.346 mA/cm^2^). *J_sc_* is mainly proportional to the light-harvesting (LHE) and electron collection (Φ_coll_) efficiencies [38,39,40]. The LHE is closely related to the light absorptivity (A) of the loaded dyes, i.e., LHE = 1–10^–A^ [39,40], and therefore can be estimated by measuring the amount of dyes loaded on the ZnO surface. Thus, to investigate the influence of the LHE on the *J_sc_* enhancement, we calculated the amount of dye loaded on the ZnO nanorod surface on the basis of the Beer-Lambert equation and the absorbance of dyes [4,32]. As displayed in Figure 6, Table 3 and Table S4 of the ESI, the average dye-loading quantities for the pristine ZnO/FTO and Ag^+^(20)–ZnO/FTO photoelectrodes were 3.07 × 10^–4^ and 6.51 × 10^–4^ mol/cm^3^, respectively. The quantity of dye molecules adsorbed on Ag^+^(20)–ZnO/FTO increased by approximately 210% compared with that of pristine ZnO/FTO. We attributed the higher dye-loading amount in the Ag^+^(20)–ZnO/FTO to the electrostatic attraction between Ag^+^–deposited ZnO and carboxylate anions (–COO^−^) of N719 dyes. A similar phenomenon was reported by Ko et al. [11]. They revealed that the H^+^-deposited TiO_2_ induced high electrostatic attraction between the positively charged TiO_2_ and the carboxylate anions of the dyes. The higher electrostatic attraction led to an increase in the collision frequency of the ruthenium dye with adsorption sites on TiO_2_, resulting in a much faster sensitization rate. Considering that greater amount of dyes adsorbed on the ZnO electrodes could generate more excitons, the electrostatic adsorption of dyes could increase the LHE. Overall, the electrostatic sensitization method was effective in increasing the dye-loading amount within a short time (20 min), thus leading to an improvement in *J_sc_*. Meanwhile, as the deposition time of silver ions was extended over 30 min, the dye-loading amounts were rather reduced. This could be because excessive silver ions deposited on the ZnO nanorod surface occupied dye adsorption sites on the ZnO nanorods, resulting in a sharp reduction in both *J_sc_* and *V_oc_* as shown in Figure 4.

**Figure 7:**
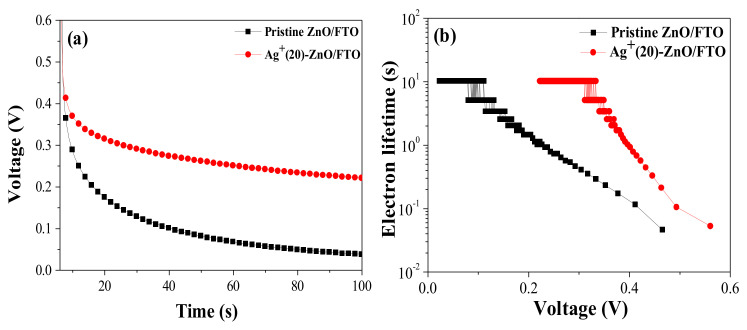
(**a**) OCVD Curves and (**b**) the electron lifetime versus the *V_oc_* for the DSCs with the pristine ZnO/FTO and Ag^+^(20)–ZnO/FTO.

The Φ_coll_ value can be estimated from the lifetime of the electrons injected from the photoexcited dyes, i.e., a longer electron lifetime can enhance the Φ_coll_ value [41]. As presented in Figure 7, we could calculate the electron lifetime (τ) of DSCs with the pristine ZnO/FTO and Ag^+^(20)–ZnO/FTO electrodes using OCVD measurement results Figure 7a and Equation (1); where *k* is the Boltzmann constant, *T* is the temperature, *e* is the electron charge, and *dV_oc_/dt* is the derivative of the *V_oc_* transient [42,43].

(1) $$\tau =-\frac{kT}{e}{\left(\frac{dVoc}{dt}\right)}^{-1}$$

Much longer electron lifetime was observed in the DSC with Ag^+^(20)–ZnO/FTO, compared to that of the control cell with the pristine ZnO/FTO electrode, as shown in Figure 7b, indicating that back electron transfer in the DSCs with the Ag^+^(20)–ZnO/FTO electrode was retarded, and the injected electrons survived in the ZnO conduction band. The increased electron lifetime in the device with the Ag^+^(20)–ZnO/FTO was due to the increase in the dye-loading quantity on the ZnO nanorod surface. When the sensitization was electrostatically conducted, the ZnO surface was effectively covered with the N719 dye. The dyes prevent direct contact of the electrolyte ions (I_3_^–^) to the ZnO surface, thus reducing the back electron transfer between I_3_^–^ in the electrolyte and electrons in the ZnO conduction band [chemical reaction (2)]. This could extend the lifetime of the electrons injected from the sensitized dyes.

I_3_^−^ + 2e^−^ (ZnO)→3I^–^ (2)

Thus, OCVD studies showed that the back electron transfer was retarded, which was attributed to the larger amounts of dyes adsorbed on the ZnO nanorod surface, resulting in an enhanced electron lifetime. This can contribute to an increase in Φ_coll_. Overall, the high *J_sc_* value in the DSC with the Ag^+^(20)–ZnO/FTO was due to the enhancement in both the LHE and Φ_coll_, resulting from the increased dye-loading amounts compared with those of the control device with the pristine ZnO/FTO.

#### 3.3.3. Influences of Ag^+^ Deposition on the *V_oc_*

An increment in *V_oc_* value (0.675 V) was also observed in the champion DSC with the Ag^+^(20)–ZnO/FTO, compared to that of the control device (0.662 V). This can be explained by the longer lifetime of electrons [44]. The *V_oc_* value is given by Equation (3) [45,46], where *k* and *T* are the same as those described in Equation (1), I_inj_ is the flux of the charge resulting from the sensitized injection, and *n_cb_* is the concentration of electrons at the ZnO surface. *k_et_* and [I_3_^–^] are the rate constants for the reduction [chemical reaction (2)] of I_3_^−^ by the conduction band electrons and the concentration of I_3_^−^, respectively.

(3) $$V_{oc}=\frac{kT}{e}\;\ln\left(\frac{I_{\mathrm{inj}}}{n_{cb}\;k_{et}\left[I_{3}^{-}\right]}\right)$$

Equation (3) indicates that *V_oc_* and *k_et_* are inversely correlated, and *k_et_* can be conjectured by measuring the electron lifetime. A longer electron lifetime means a lower *k_et_* value, i.e., a lower possibility to proceed the chemical Equation (2). As mentioned previously (OCVD studies), it turned out that back electron transfer at the interface of ZnO and electrolyte was retarded when the Ag^+^(20)–ZnO/FTO was applied to the photoelectrode of DSC. This fact suggests that the *k_et_* in Equation (3) decreases. Thus, the back electron transfer was delayed by the use of the Ag^+^(20)–ZnO/FTO, which resulted in a decreased *k_et_*, therefore leading to an improvement in *V_oc_* based on Equation (3).

#### 3.3.4. Influences of Ag^+^ Deposition on the *FF*

**Figure 8:**
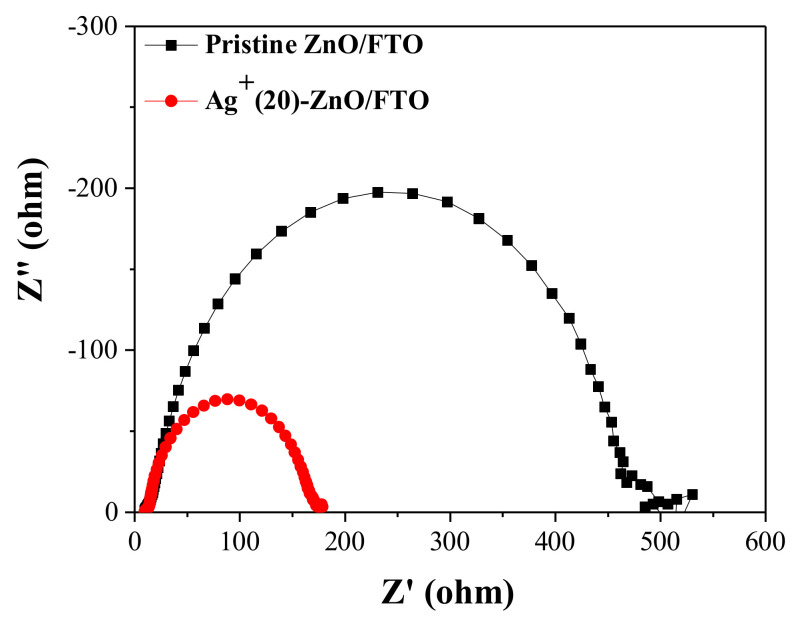
Nyquist plots of EIS spectra for the DSCs with the pristine ZnO/FTO and the Ag^+^(20)–ZnO/FTO, measured under illumination (AM 1.5 condition).

The champion cell with the Ag^+^(20)–ZnO/FTO showed a higher *FF* value (50.14%) compared with that of the control device with the pristine ZnO/FTO (40.52%). *FF* value is affected by the series (*R_s_*) and shunt (*R_sh_*) resistances of DSCs, i.e., lower *R_s_* and/or higher *R_sh_* can increase it [47,48]. From the slope of the *J*–*V* curves at *V_oc_* and *J_sc_*, we could obtain the *R_s_* and *R_sh_* values, respectively [49]. As shown in Table 4, a lower *R_s_* value (37.6 Ωcm^2^) in the champion cell with the Ag^+^(20)–ZnO/FTO was recorded compared with that of the control device with the pristine electrode (72.3 Ωcm^2^). The *R_s_* value is closely related to the internal resistance of the solar cell and can be partially estimated via EIS measurements. As shown in Figure 8, interfacial resistance at the ZnO/N719/electrolyte was reduced to approximately 170 Ω from 460 Ω for the control cell with the pristine ZnO/FTO, contributing to a decrease in *R_se_*. Meanwhile, the *R_sh_* of the device with the Ag^+^(20)–ZnO/FTO is increased by 1532.8 Ωcm^2^ compared with that of the control device (700.4 Ωcm^2^). *R_sh_* is affected by back electron transfer and leakage current, i.e., back electron transfer can decrease the *R_sh_* value in solar cells [47]. As depicted earlier, the back electron transfer at the ZnO/electrolyte interface was delayed by the Ag^+^ deposition on the surface of the ZnO nanorods, indicating that the *R_sh_* value in the DSC with the Ag^+^(20)–ZnO/FTO would be larger than that of the control device with the pristine ZnO/FTO. Thus, it was evident that the lowered *R_s_* and elevated *R_sh_* led to the increase in *FF*.

### 3.4. Stability of DSCs with Ag^+^(20)–ZnO/FTO

It is necessary to examine the effects of silver ions deposited on ZnO nanorod surfaces on the stability of DSCs. We first confirmed whether silver ions would be detached from the ZnO surface when they contacted with electrolytes. Commercial ZnO powder was used for EDS analysis, because we could not obtain enough amounts of ZnO nanorods by the CBD method. To prepare silver-ion-deposited ZnO [Ag^+^(20)–ZnO], ZnO particles (8 g) were soaked into an aqueous AgNO_3_ solution (50 mM, 200 mL) for 20 min to deposit Ag^+^, rinsed and dried. The Ag^+^(20)–ZnO particles were subsequently dipped in commercial I^–^/I_3_^–^-based electrolytes for 720 h. It was confirmed from EDS analyses that the weight ratio of silver was 0.12% in as-prepared Ag^+^(20)–ZnO particle, almost similar to that (0.17%) in 720 h-soaked Ag^+^(20)–ZnO, as shown in Figures S6 and S7 of the ESI. This implies that silver ions deposited ZnO surfaces do not contaminate the I^–^/I_3_^–^-based electrolytes.

**Figure 9:**
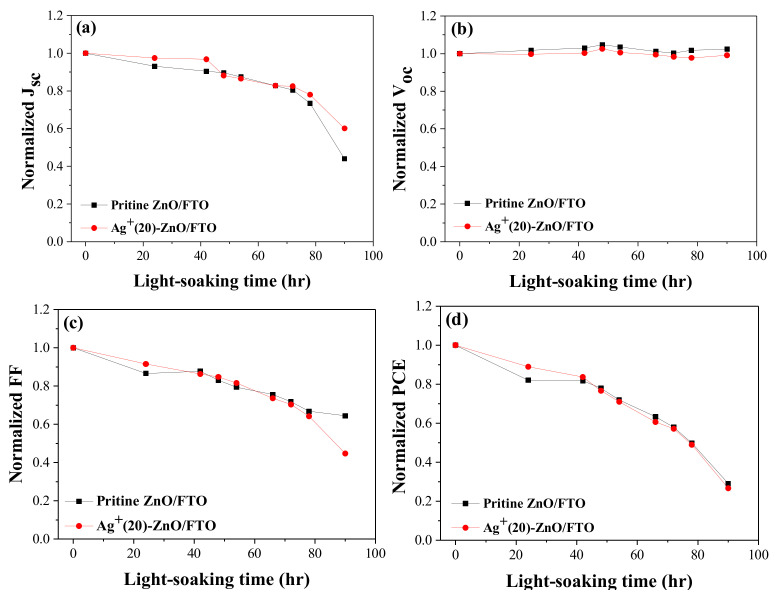
Light-soaking stability; (**a**) *J_sc_*, (**b**) *V_oc_*, (**c**) *FF*, and (**d**) PCE of DSCs with the pristine ZnO/FTO and Ag^+^(20)–ZnO/FTO. Photovoltaic parameters were measured under illumination (AM 1.5 condition).

We compared the light-soaking stability of DSCs by measuring their photovoltaic parameters over time. The prepared ZnO-based DSCs were additionally sealed using hot-melt glue sticks to minimize electrolyte leakage. Figure 9 illustrates the time-dependent performance variations of the cells under A.M 1.5 global illumination at room temperature. Light-soaking stability of DSC with the Ag^+^-deposited ZnO nanorod was identical to that of the control cell with the pristine ZnO, indicating that silver ions did not affect the device stability.

## 4. Conclusions

We formed vertically aligned ZnO nanorods on FTO glasses at a growth temperature of 75 °C, and they were applied to the photoelectrodes of DSCs. When the ZnO nanorods were grown for 8 h, DSC showed a PCE value of 0.629% with 2.346 mA/cm^2^ of *J_sc_*, 0.662 V of *V_oc_,* and 40.52% of *FF*. By replacing the pristine ZnO/FTO electrode with the silver-ion-deposited ZnO nanorods [Ag^+^(20)–ZnO/FTO], the PCE of the cell was considerably improved up to 1.138% (*J_sc_* = 3.362 mA/cm^2^, *V_oc_* = 0.675 V, and *FF* = 50.14%). This increase in the PCE was attributed to a significant enhancement in the dye-loading amount due to the electrostatic attraction between the positively charged ZnO and carboxylate anions (–COO^−^) of the N719 dyes. This indicates that electrostatic dye adsorption is an effective method to increase the dye-loading amount in a short sensitization time (typically 20 min), thus reducing the formation of the Zn^2+^/dye complex, leading to enhanced photovoltaic performance.

## Figures and Tables

**Table 1:** Reported methods for improving dye-loading amount on the ZnO surface.

Structure of ZnO	Method for Increasing Dye-Loading Amount	Improvement Ratio (%)		Ref.
		Dye-Loading Amount	PCE	
Nanoparticle	Ti doping	168 (1.10→ 1.85 × 10^−6^ mol/cm^2^)	145 (3.82 → 5.56%)	[21]
Nanorod	Optimization of aspect ratio	153 (3.36→ 5.13 × 10^−6^ mol/dm^3^)	171 (0.55 → 0.94%)	[22]
Nanorod	Optimization of heat treatment temperature	134 (2.88→ 3.85 × 10^−6^ mol/cm^3^)	133 (0.43 → 0.57%)	[23]
Nanorod	Decoration with ZnO nanofoil	230 (0.195→ 0.448) ^a^	163 (1.20 → 1.95%)	[24]
Nanorod	Electrostatic attraction	210 (3.07→ 6.51 × 10^−4^ mol/cm^3^)	180 (0.629 → 1.138%)	This study

^a^ absorbance of dyes desorbed from ZnO nanorod surface.

**Table 2:** Photovoltaic parameters and dye-loading amounts of DSCs with growth time of ZnO nanorods.

Growth Time (h)	*J_sc_* (mA/cm^2^)	*V_oc_* (V)	*FF* (%)	*PCE* (%)	Adsorbed Dye (10^–4^ mol/cm^3^)
4	0.844 ± 0.199	0.655 ± 0.013	38.73 ± 3.60	0.214 ± 0.055	0.93 ± 0.29
6	1.425 ± 0.107	0.651 ± 0.008	40.49 ± 1.87	0.377 ± 0.045	1.66 ± 0.23
8	2.077 ± 0.297	0.651 ± 0.009	41.13 ± 0.95	0.557 ± 0.089	3.07 ± 0.35
10	1.236 ± 0.475	0.637 ± 0.018	41.71 ± 3.94	0.320 ± 0.123	1.16 ± 0.25

**Table 3:** Photovoltaic parameters and dye-loading amounts of DSCs with deposition time of silver ions on 8 h-grown ZnO nanorods.

Deposition Time (min)	*J_sc_* (mA/cm^2^)	*V_oc_* (V)	*FF* (%)	PCE (%)	Adsorbed Dye (10^–4^ mol/cm^3^)
0	2.077 ± 0.297	0.651 ± 0.009	41.13 ± 0.95	0.557 ± 0.089	3.07 ± 0.35
10	2.310 ± 0.125	0.685 ± 0.014	52.40 ± 3.20	0.830 ± 0.074	3.68 ± 0.69
20	2.784 ± 0.504	0.674 ± 0.021	52.10 ± 2.89	0.973 ± 0.144	6.51 ± 0.85
30	0.659 ± 0.562	0.549 ± 0.062	50.64 ± 2.60	0.189 ± 0.170	2.64 ± 0.49
40	0.802 ± 0.349	0.513 ± 0.013	51.70 ± 3.24	0.210 ± 0.082	1.80 ± 0.22

**Table 4:** Photovoltaic parameters of the champion cells with the pristine ZnO/FTO and Ag^+^(20)–ZnO/FTO electrodes.

Photoelectrodes	*J_sc_* (mA/cm^2^)	*V_oc_* (V)	*FF* (%)	*PCE* (%)	*R_s_* (Ωcm^2^)	*R_sh_* (Ωcm^2^)
Pristine ZnO/FTO	2.346	0.662	40.52	0.629	72.3	700.4
Ag^+^(20)–ZnO/FTO	3.362	0.675	50.14	1.138	37.6	1532.8

## Data Availability

The data presented in this study are available on request from the corresponding author.

## Supplementary Materials

The following are available online at https://www.mdpi.com/article/10.3390/nano12030372/s1, Figure S1: Chemical structure (a) and UV-visible absorption spectrum (b) of N719 dye. Ethanolic N719 solution was used for the absorption spectrum measurement, Figure S2: Schematic energy band diagram of ZnO-based DSC, Figure S3: SEM image (top view) of ZnO nanorods formed on a FTO glass, Figure S4. XRD pattern of ZnO nanorods formed on a FTO glass. Table S1: Averages and standard deviations of cell parameters, which were measured using 3–4 cells with ZnO nanorods, Table S2: Amounts of N719 dyes loaded on the surface of ZnO nanorods with growth time, Figure S5: XPS survey spectrum of Ag^+^(20)–ZnO/FTO electrode, Table S3: Averages and standard deviations of cell parameters, which were measured using 3–4 cells with the Ag^+^–ZnO/FTO electrodes, Table S4: Amounts of N719 dyes loaded on the surface of 8 h-grown ZnO nanorods with deposition time of silver ions, Figure S6: EDS spectra of (a) as-prepared and (b) 720-h-soaked Ag^+^(20)-ZnO particles, Figure S7: SEM (left) and EDS mapping (right) images of (a) as-prepared and (b) 720-h-soaked Ag^+^(20)-ZnO particles.
