# Supplementary material for: Enhancement in Photovoltaic Performance of Solar Cells by Electrostatic Adsorption of Dyes on ZnO Nanorods
Source: Nanomaterials (Basel). 2022 Jan 24;12(3):372. doi: 10.3390/nano12030372 (PMC8838287; doi:10.3390/nano12030372)
Supplement: Supplementary file 1 [file nanomaterials-12-00372-s001.zip › nanomaterials-1535331-supplementary.pdf]

nanomaterials

Article

Enhancement in Photovoltaic Performance of Solar Cells by Electrostatic Adsorption of Dyes on ZnO Nanorods

Seong Il Cho [1,†], Baekseo Choi [1,†], Byeong Chul Lee [1], Yunsung Cho [2] and Yoon Soo Han [1,*]

1 School of Advanced Materials and Chemical Engineering, Daegu Catholic University, Gyeongbuk 38430, Korea; pokw1231@naver.com (S.I.C.); skdiwlsdnr@naver.com (B.C.); bclee@live.co.kr (B.C.L.)
2 School of Electronic and Electrical Engineering, Daegu Catholic University, Gyeongbuk 38430, Korea; philos@cu.ac.kr
* Correspondence: yshancu@cu.ac.kr; Tel.: +82-53-850-2773; Fax: +82-53-359-6662
† These authors contributed equally to this work.

1. Materials for fabrication of ZnO-based DSCs

Commercial FTO glass with a sheet resistance of 7 Ω/square (TCO22–7), N719 dye (Ruthenizer 535–bisTBA), hot-melt adhesive (SX1170–60PF, Surlyn), and I^-/I_3^--based electrolytes (AN–50) were purchased from Solaronix (Aubonne, Switzerland). Zinc acetate dihydrate, zinc nitrate hexahydrate, and hexamethylenetetramine (Sigma–Aldrich Co.; St. Louis, MO, USA) were used to form the ZnO nanorods Platinum paste (PT–1, Dyesol–Timo JV; Seoul, Korea) was chosen as the source for the Pt counter electrode. All the chemicals were used without further purification.

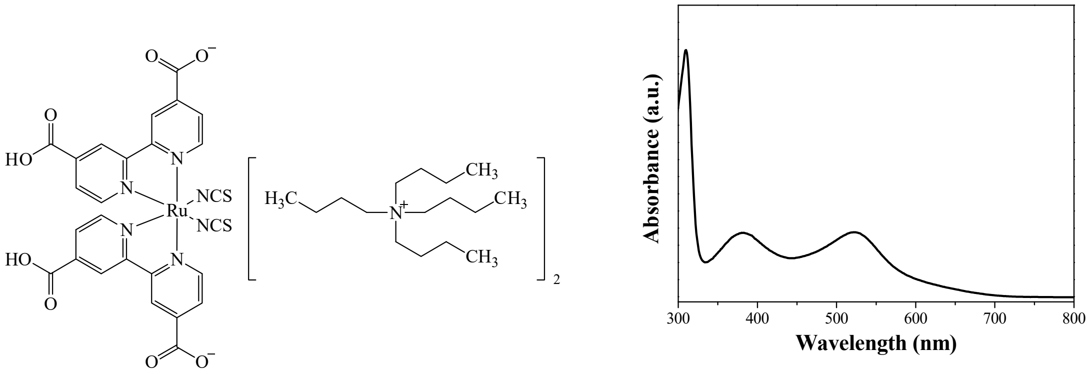

(a) (b)

Figure S1. Chemical structure (a) and UV-visible absorption spectrum (b) of N719 dye. Ethanolic N719 solution was used for the absorption spectrum measurement.

2. Detailed fabrication conditions and energy band diagram of ZnO-based DSC

To prepare the working electrodes, the FTO glasses were cleaned in a detergent solution with sonication for 20 min, and then thoroughly rinsed with deionized (DI) water and ethanol. To deposit a seed layer, we spin-coated a 5 mM solution of zinc acetate dihydrate in ethanol on the cleaned FTO glasses at a speed of 3000 rpm for 30 s and then dried them on a preheated hot plate at 150 °C for 15 min. After this process was repeated three times, the glasses were annealed at 350 °C for 15 min. The FTO glass with the seed layer was immersed in an aqueous solution of zinc nitrate hexahydrate (35 mM) and hexamethylenetetramine (35 mM) at 75 °C for 4–10 h to grow ZnO nanorods on the FTO substrate.

The ZnO nanorods on the FTO glass were rinsed with DI and ethanol and then annealed at 450 °C for 30 min to produce ZnO/FTO photoelectrodes. The deposition area of ZnO was adjusted by masking using Kapton tape. The ZnO/FTO photoelectrodes were soaked in an aqueous solution (50 mM) of silver nitrate for 0–40 min to deposit silver ions onto the ZnO surfaces. Subsequently, the resulting electrodes were rinsed with water and ethanol and then dried at 65 °C for 10 min to produce silver-ion-deposited electrodes (Ag^+–ZnO/FTO). The pristine ZnO/FTO and Ag^+–ZnO/FTO photoelectrodes were separately immersed into 0.5 mM of ethanolic N719 dye solution for 20 min to obtain the working electrodes.

To prepare the counter electrodes, two holes were formed in the FTO glasses using a drill, and the glasses were cleaned using the method described above. Pt layers were formed on the FTO glass via the doctor blade coating method using Pt paste, followed by calcination at 400 °C for 30 min. The thermally treated platinum counter electrodes were then placed on the working electrodes and sealed using a 60-μm-thick sealing material. The electrolyte was introduced into the cells through one of the two small holes to produce ZnO-based DSCs with a 25 mm^2 active area.

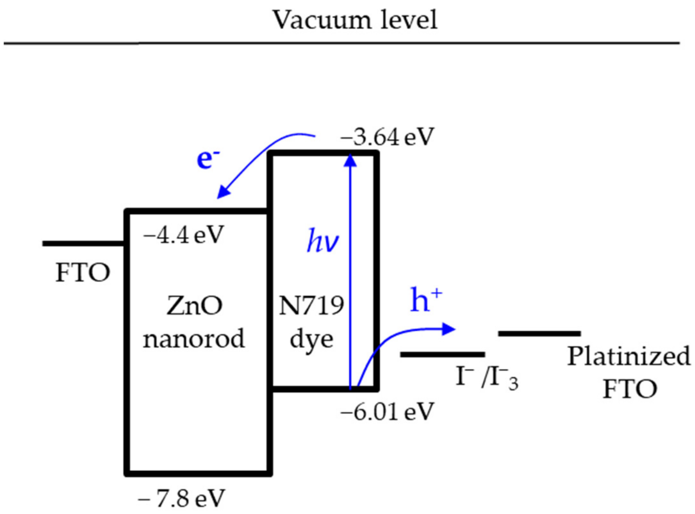

Figure S2. Schematic energy band diagram of ZnO-based DSC.

3. Instrumental measurements

Field-emission scanning electron microscopy (FE–SEM; S–4800, Hitachi High–Technology; Tokyo, Japan) equipped with energy-dispersive X-ray spectroscopy (EDS; Horiba EX-250) was used to observe the morphology of the ZnO nanorods and to examine the weight ratio of silver ions deposited on ZnO surfaces. X-ray photoelectron spectroscopy (XPS) was performed using a VG Multilab ESCA 2000 (ThermoVG Scientific, Needham, MA, USA) instrument with Mg Kα radiation. The C 1s photoelectron peak (binding energy of 284.6 eV) was used as an energy reference. For the structural characterization of the ZnO nanorods, X-ray diffraction (XRD) patterns were recorded on a X-ray diffractometer (XRD) (Empyrean, Malvern Panalytical Ltd.; Malvern, United Kingdom) in a 2θ range from 10 to 100° with a 0.026° step using CuKα radiation (1.5406 Å). The photocurrent–voltage measurements were performed using a CompactStat potentiostat (Ivium Technologies B.V.; Eindhoven, The Netherlands) and a PEC–L01 solar simulator system equipped with a 150 W xenon arc lamp (Peccell Technologies, Inc.; Yokohama, Japan). The light intensity was adjusted to 1 sun (100 mW/cm^2) using a silicon photodiode (PEC–SI01, Peccell Technologies, Inc.; Yokohama, Japan). The UV–vis absorption spectra were obtained using a SINCO NEOSYS–2000 spectrophotometer (Seoul, Korea). Open-circuit voltage decay (OCVD) measurements and electrochemical impedance spectroscopic (EIS) analyses were performed using an electrochemical analyzer (CompactStat, Ivium Technologies

B.V.; Eindhoven, The Netherlands). The active areas of the dye-adsorbed ZnO films were estimated using a digital microscope camera (SZ61, LYMPUS Corporation; Tokyo, Japan) equipped with image analysis software.

4. Morphology and characterization of ZnO nanorods

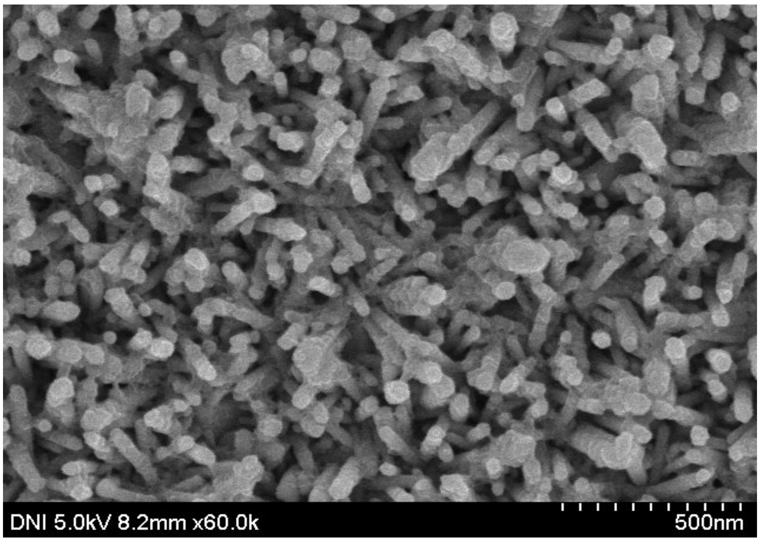

Figure S3. SEM image (top view) of ZnO nanorods formed on a FTO glass.

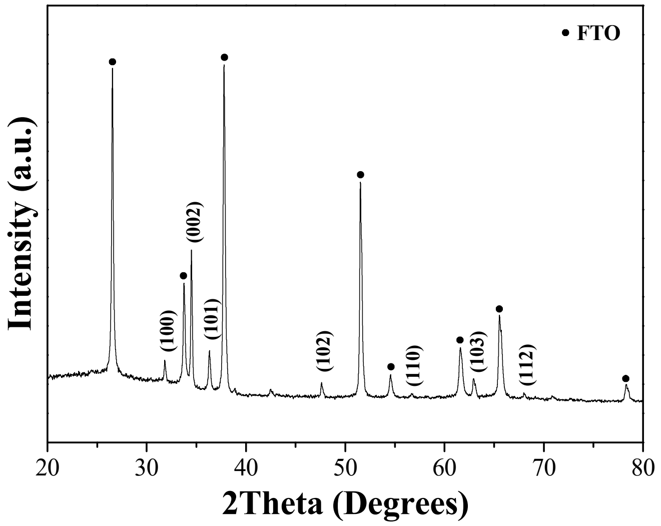

Figure S4. XRD pattern of ZnO nanorods formed on a FTO glass.

5. Photovoltaic performance of DSCs with ZnO nanorods

Table S1. Averages and standard deviations of cell parameters, which were measured using 3–4 cells with ZnO nanorods.

Growth time (hr)	J_{sc} (mA/cm^2)	V_{oc} (V)	*FF* (%)	*PCE* (%)
4	1.104	0.672	37.67	0.280

	0.805	0.642	34.79	0.180
	0.846	0.650	43.44	0.239
	0.622	0.656	39.00	0.159
	0.844±0.199	0.655±0.013	38.73±3.60	0.214±0.055
6	1.484	0.648	40.94	0.394
	1.507	0.660	42.45	0.422
	1.270	0.654	37.95	0.315
	1.439	0.642	40.60	0.375
	1.425±0.107	0.651±0.008	40.49±1.87	0.377±0.045
8	2.346	0.662	40.52	0.629
	2.254	0.646	42.53	0.619
	1.678	0.642	40.56	0.437
	2.031	0.654	40.90	0.543
	2.077±0.297	0.651±0.009	41.13±0.95	0.557±0.089
10	1.781	0.624	44.27	0.458
	1.018	0.630	43.69	0.280
	0.908	0.658	37.17	0.222
	1.236±0.475	0.637±0.018	41.71±3.94	0.320±0.123

6. Dye-loading amounts with growth time of ZnO nanorods

Table S2. Amounts of N719 dyes loaded on the surface of ZnO nanorods with growth time**.**

Growth time (hr)	Adsorbed dye (10^{-4} mol/cm^3)
4	1.11
	1.09
	0.60
	0.93±0.29
6	1.39
	1.80
	1.79
	1.66±0.23
8	3.45
	2.97
	2.78
	3.07±0.35
10	1.42
	1.14
	0.93
	1.16±0.25

7. Characterizations of silver-ion-deposited ZnO nanorods

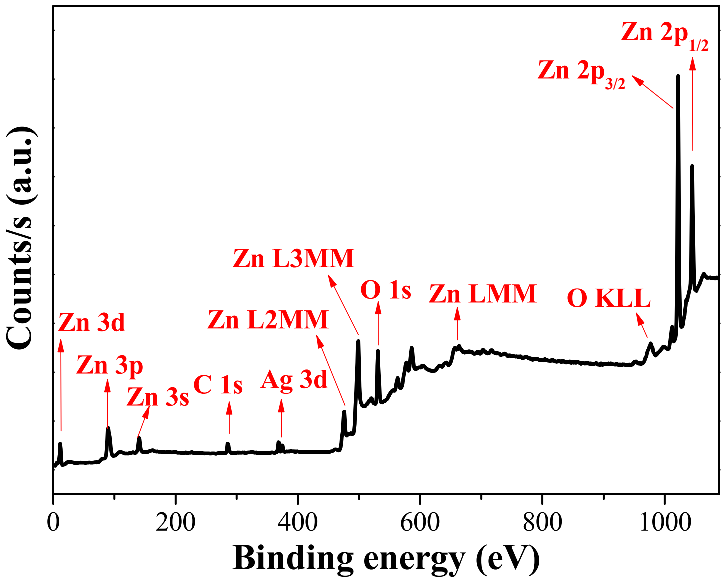

Figure S5. XPS survey spectrum of Ag^{+}(20)–ZnO/FTO electrode.

8. Photovoltaic performance of DSCs with Ag^{+}-ZnO/FTO

Table S3. Averages and standard deviations of cell parameters, which were measured using 3–4 cells with the Ag^{+}–ZnO/FTO electrodes.

Deposition time (min)	J_{sc} (mA/cm^2)	V_{oc} (V)	*FF* (%)	*PCE* (%)
0	2.346	0.662	40.52	0.629
	2.254	0.646	42.53	0.619
	1.678	0.642	40.56	0.437
	2.031	0.654	40.90	0.543
	2.077±0.297	0.651±0.009	41.13±0.95	0.557±0.089
10	2.430	0.676	53.93	0.887
	2.180	0.702	48.73	0.746
	2.320	0.678	54.55	0.858
	2.310±0.125	0.685±0.014	52.40±3.20	0.830±0.074
20	2.560	0.694	50.74	0.901
	3.362	0.675	50.14	1.138
	2.430	0.652	55.42	0.879
	2.784±0.505	0.674±0.021	52.10±2.89	0.973±0.144
30	1.270	0.620	47.68	0.377
	0.165	0.522	51.67	0.047
	0.542	0.506	52.56	0.144
	0.659±0.562	0.549±0.062	50.64±2.60	0.189±0.170

40	0.754	0.522	51.92	0.205
	1.172	0.520	48.36	0.295
	0.479	0.498	54.82	0.131
	0.802±0.349	0.513±0.013	51.70±3.24	0.210±0.082

9. Dye-loading amounts with deposition time of silver ions on ZnO nanorods

Table S4. Amounts of N719 dyes loaded on the surface of 8hr-grown ZnO nanorods with deposition time of silver ions**.**

Deposition time (min)	Adsorbed dye (10^{-4} mol/cm^3)
0	3.45
	2.97
	2.78
	3.07±0.35
10	3.91
	4.22
	2.91
	3.68±0.69
20	6.71
	7.24
	5.58
	6.51±0.85
30	2.60
	3.15
	2.18
	2.64±0.49
40	1.92
	1.55
	1.93
	1.80±0.22

10. EDS spectra and mapping images of Ag^+(20)–ZnO particles

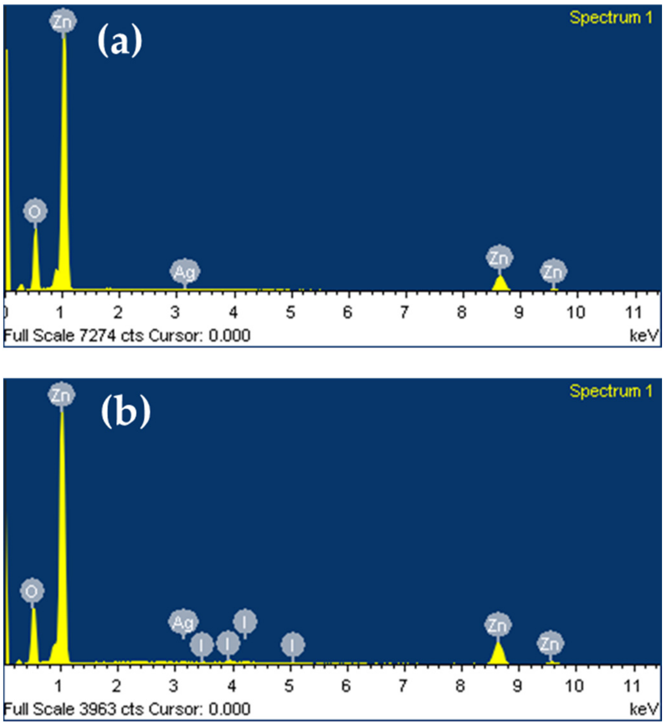

Figure S6. EDS spectra of (a) as-prepared and (b) 720-h-soaked Ag^{+}(20)-ZnO particles.

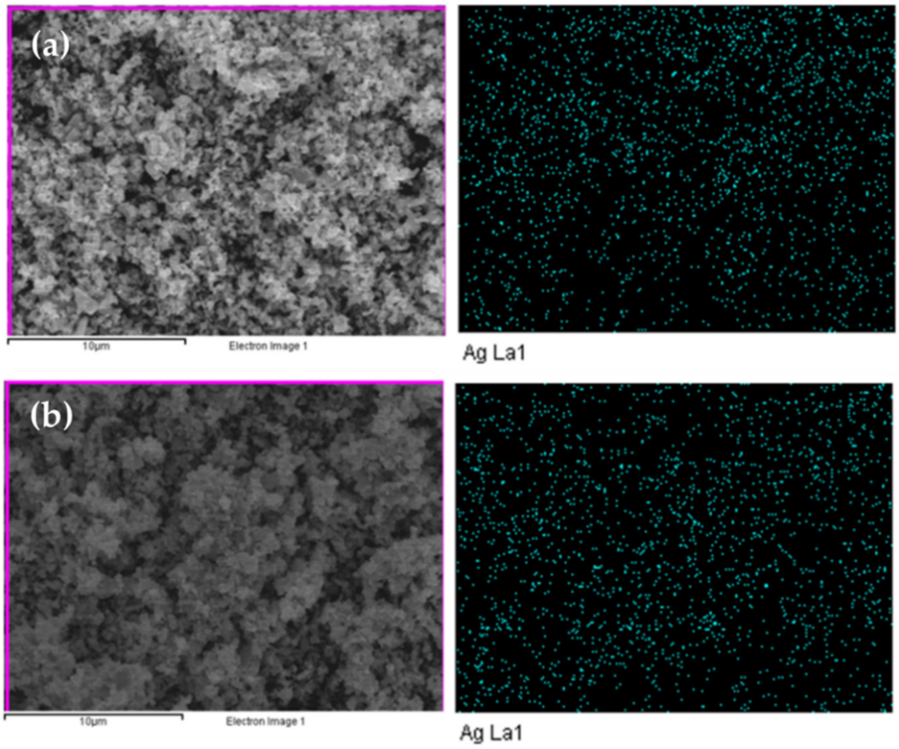

Figure S7. SEM (left) and EDS mapping (right) images of (a) as-prepared and (b) 720-h-soaked Ag^{+}(20)-ZnO particles.
